# Plasticity in Sexual Dimorphism Enhances Adaptation of Dioecious *Vallisneria natans* Plants to Water Depth Change

**DOI:** 10.3389/fpls.2019.00826

**Published:** 2019-07-03

**Authors:** Yin Zhou, Lei Li, Zhiping Song

**Affiliations:** ^1^Ministry of Education Key Laboratory for Biodiversity Science and Ecological Engineering, Institute of Biodiversity Science, Fudan University, Shanghai, China; ^2^Jiangxi Province Key Laboratory of Watershed Ecosystem Change and Biodiversity, Center for Watershed Ecology, Institute of Life Science and School of Life Sciences, Nanchang University, Nanchang, China; ^3^Key Laboratory of Poyang Lake Environment and Resource Utilization, Ministry of Education, Nanchang University, Nanchang, China; ^4^National Ecosystem Research Station of Jiangxi Poyang Lake Wetland, Nanchang, China

**Keywords:** adaptive strategy, dioecy, life history trait, phenotypic plasticity, photosynthesis, sexual dimorphism, submerged macrophytes, water depth gradient

## Abstract

Sexual dimorphism in vegetative and reproductive traits is associated with contrasting strategies of males and females for response to varied environmental conditions, causing sex-specific reproduction success and consequently long-distance dispersal and colonization. Aquatic plants usually exhibit rich phenotypic plasticity and great diversity in reproductive systems, but the influence of aquatic conditions on the plasticity of sexual dimorphism has received less attention. Using a common garden experiment with dioecious submerged plant *Vallisneria natans* grown at various water depths simulating different light availability, we measured variations in 20 traits for females and 19 traits for males (total = 540 plants from 30 seed families) including morphology, reproductive traits and photosynthesis. We investigated sex-specific plastic responses and variation of sexual dimorphism in response to water depth change. Females displayed much greater leaf length, vegetative biomass and resource allocation to reproduction than males at all depths, whereas spathe number and gamete production per spathe displayed reverse pattern. Besides most traits in each sex (16 in female and 12 in male) showing striking phenotypic plasticity, the degree of sexual dimorphism increased significantly for total biomass and reproductive investment, but decreased for leaf length, spathe number and flowering ramet percentage in low light and deep water. Females varied more than males in leaf length, total biomass, reproductive investment, length and biomass of reproductive organs and rate of photosynthesis in response to decreased underwater light availability, suggesting that female has greater plasticity than male. These findings illustrated considerable plasticity in the degree of sexual dimorphism in a variety of vegetative and reproductive traits across different environments driven by the contrasting reproductive functions of the sexes in relation to pollen and seed dispersal. Females of *V. natans* responded more plastically than males to low light conditions resulted from water depth variation in either aboveground vegetative growth or reproduction. This study provides novel insight into adaptive strategies of submerged dioecious macrophytes to survive and increase fitness in freshwater habitats.

## Introduction

The trade-offs between reproduction and somatic growth is the most basic hypothesis of life history theory in plants (Obeso, 2002). Among dioecious plants, male and female sexual functions occur on separate plants and females usually bear higher costs of reproduction than males because of seed and fruit production (Delph, 1999; Barrett and Hough, 2013; Hultine et al., 2016). The differences in reproductive costs due to different investment in reproduction between sexes will bring about sexual dimorphisms. For example, male and female plants often show sex-specific difference in morphological and physiological traits, survival, defense and herbivory and patterns of growth and resource allocation (Geber et al., 1999; Cornelissen and Stiling, 2005; Barrett and Hough, 2013). Males and females optimize their fitness, respectively, by evolving divergent life history traits, probably permitting sex-specific resource acquisition strategies. Nevertheless, similar performances of the sexes have also been reported in a range of dioecious terrestrial species (see references in Nicotra, 1999; Obeso, 2002), such as *Buchloe dactyloides* (Quinn, 1991) and *Carex picta* (Delph et al., 1993).

For dioecious plants, differing reproductive costs can cause the sexes to respond to environmental conditions in different ways, which can cause variation in the degree of sexual dimorphism along environmental gradients (Delph and Bell, 2008). In resource limited environments, reduction in resource allocation to reproduction might be more marked in females than in males (Dawson and Geber, 1999; Pickering and Hill, 2002), because females generally incur higher costs for a given reproductive period. Due to the higher cost of reproduction, females might be expected to be generally more responsive to environmental conditions in terms of vegetative growth than males (Dawson and Geber, 1999; Delph, 1999). Therefore, the sexual differences in aboveground vegetative growth increased in more stressful environments for several terrestrial species (Dawson and Ehleringer, 1993; Eckhart and Chapin, 1997). However, some studies did not show clear variation in sexual dimorphism in either reproductive allocation or vegetative growth between the sexes (Sakai et al., 2006; Hesse and Pannell, 2011; Teitel et al., 2016). A growing body of work has tested for variation in sexual dimorphism in terrestrial plants under different environmental conditions with mixed results. As far as we are aware, how aquatic conditions influence the plasticity in sexual dimorphism in dioecious macrophytes is still mostly unknown (but see Li et al., 2019a). Studies of variation of sexual dimorphism under different conditions could provide insights into our understanding of the adaptive strategies of dioecious macrophytes to environmental changes.

Aquatic plants are a fascinating group for both naturalists and plant biologists, and usually exhibit rich phenotypic plasticity and great diversity in their reproductive systems (Eckert et al., 2016). Dioecy occurs frequently in aquatic macrophyte families, especially those that are submersed (Les, 1988). Aquatic plants often occupy habitats characterized by strong environmental gradients, providing an excellent opportunity for examining plasticity in sexual dimorphism. Water depth is a major environmental factor influencing the distribution, growth and reproduction of submerged macrophytes, which are greatly sensitive to water level fluctuation (Chambers and Kaiff, 1985; Fu et al., 2014; Ye et al., 2018). An increase in water depth reduces the light penetration to the lake bottom, especially in eutrophic water (Zhang et al., 2016). Many studies have examined the physiological and morphological response in vegetative structures (phenotypic plasticity) of submerged macrophytes to varying water depths (Maberly, 1993; Strand and Weisner, 2001; Zhou et al., 2017). Submerged macrophytes normally adapt to low light availability in water column by one of two distinct strategies – elongating shoot toward water surface to alleviate low light stress or enhancing low light tolerance through photosynthetic adjustments (Riis et al., 2012; Chen et al., 2016). Yet relatively fewer studies exist dealing with the variation in plant reproductive traits (Li et al., 2017, 2018), and even fewer studies have been conducted to further understand patterns of sexual dimorphism in dioecious macrophytes compared to their terrestrial relatives (Dorken and Barrett, 2004; Li et al., 2019b). Reproductive strategies influence the response of populations to environmental variation (Dorken and Barrett, 2004). For example, plasticity in sexual system of dioecious plants has important roles in regulating mating opportunities and ensuring reproduction in stressful conditions, thus influencing the ability of population to disperse over long distances, colonize and persist in different environments. In natural waterbodies, high water depth can be presumed to be representative of resource limited environments due to low light intensity. Water depth not only induces light changes but also affect the potential of a plant to capture carbon. Therefore, integrated studies of reproductive and vegetative traits along water depth gradients are important to analyze variation in sexual dimorphism of submerged macrophytes in response to environmental differences.

In this study, the overall aim was to determine the magnitude of sexual dimorphism in a submerged dioecious macrophyte and test how the sexual dimorphism is affected by change in resources. Given the variations in sexual strategies in response to environmental change, we hypothesize that significant sexual dimorphism exists in the widespread submerged macrophyte *Vallisneria natans* and the degree of sexual dimorphism varies with the change in light availability, i.e., existence of plasticity in sexual dimorphism. We designed a water depth gradient simulating light availability to measure 20 and 19 traits in males and females of *V. natans*, respectively (16 was common between sexes). Especially, we want to answer these questions: (1) what is the magnitude of sexual dimorphism in *V. natans* across 16 common traits?; (2) do sexual dimorphisms plastically vary with increasing water depth?; (3) do adaptive strategies (e.g., change in trait expression) to reduced light availability vary between sexes?

## Materials and Methods

### Plant Species

*Vallisneria natans* (Lour.) Hara (Hydrocharitaceae), a submerged dioecious macrophyte with a wide geographical range, is a dominant native species in many freshwater habitats in China. It is frequently, though not always, annual species (Zhou et al., 2016). New rosette-like ramets are produced through extension of stolons. The spathes are located among bases of linear leaves, containing hundreds of minute (0.5 mm) male flowers and after maturation male flowers are released to the water surface. A female spathe has only one female flower with a long spiral peduncle reaching the water surface. Pollination occurs on the water surface, and after pollination the peduncles of female flowers coil tightly and retract pollinated flowers underwater, where fleshy fruits further develop. Flowering and seed set are indeterminate, occurring from July to October in the study area in eastern China. This species play important roles in freshwater ecosystems, as it can provide food for waterfowls, nursery habitats for fishes, and a substrate for invertebrates and may have great impact on water quality.

### Experimental Design

To access the magnitude of sexual dimorphism in *V. natans* across 16 common life history traits (defined as traits relevant to growth and reproduction, Tonnabel et al., 2017), and if the traits for either males or females differed with water depth, we established a common garden experiment (experiment I) along a water depth gradient at the Fudan University, Shanghai, East China. We also conducted an outdoor experiment (experiment II) involved two water depth treatments (0.3 and 1.3 m) to study sex-specific patterns of phenotypic plasticity in response to water depth (i.e., the interaction between sex and water depth).

### Experiment I: Common Garden Experiment

In November 2012, 30 intact and mature fruits were randomly sampled from 30 females (i.e., 30 families) in our experimental population of *V. natans* undergoing several years of random mating. The flesh and pectin of each fruit were carefully removed, and the seeds from the same family were pooled into a centrifuge tube (1.5 mL) and then preserved at 4°C.

In March 2013, 120 seeds of each family were germinated in plastic jars (12-cm diameter and 12-cm height) covered by 2-cm-deep sterile substrate (mixture of mud and lake sand at 4:1 volume ratio). The jars were filled with water and placed under constant room temperature at 25°C with a 12 h:12 h light cycle. In early May 2013, when the seedlings produced three or four leaves, we randomly transplanted 60 seedlings of each family into plastic pots (12-cm top diameter and 10-cm height, one seedling per pot) filled with 8-cm-deep sterile substrate. Before transplantation, plant height of each seedling was measured. In total, 1800 pots were placed randomly in the outdoor experimental pool full of tap water (TN: 0.72 ± 0.06 mg⋅L^–1^, TP: 0.034 ± 0.005 mg⋅L^–1^, mean ± SE, *n* = 3) with a water depth gradient of 0.3, 0.8, and 1.3 m, representing the shallow, moderate and deep water, respectively. Finally, there were 600 plants in each water depth treatment (20 plants from each 30 families). The water level of the pool was maintained at the highest level. The light intensity at water surface and in the water column (0.3, 0.8, and 1.3 m under the water) were 1772.4 ± 195.0, 939.2 ± 66.7, 537.8 ± 35.9, and 275.3 ± 18.3 μmol⋅m^–2^⋅s^–1^ (mean ± SE, *n* = 3), respectively, at 14:00 on sunny days in mid August, measured by a UWQ-192 sensor (Li-COR, Lincoln, NE, United States). The percentage of light availability in relation to incident light at 0.3, 0.8, and 1.3 m was 53.0, 30.3, and 15.5%, respectively. The pH and temperature in the water column were recorded at 14:00 on sunny days in mid August using a Hydrolab DS5X multi-parameter water quality analyzer (Hach Company, Loveland, CO, United States). Along water depth from shallow to deep, the pH (mean ± SE, *n* = 3) is 7.34 ± 0.158, 7.30 ± 0.153, and 7.09 ± 0.166, and the water temperature (mean ± SE, *n* = 3) is 24.4 ± 0.2, 23.8 ± 0.5, and 23.4 ± 0.4°C, respectively.

In late September 2013, at fruit maturation, we randomly selected three of well-developed male and female plants from each family for the trait measurement (90 plants of each sex for each water depth treatment). Finally, the experiment involved three water depth treatments, two sex treatments, 30 families and three replicates per treatment (a total of 270 males and 270 females). Each plant was hand-washed free of sediment and epiphytic aquatic organisms. We counted both the total and flowering ramet numbers to calculate the percentage of flowering ramets of each plant. Leaf number per flowering ramet of each plant was measured as the mean of three randomly selected flowering ramets (including the ortet) from the same plant, as well as number of spathes and fruits per flowering ramet. The total numbers of spathes per male plant and fruits per female plant were also recorded. We randomly selected three flowering ramets (including the ortet) of each plant to measure the length and width of the longest intact leaf. Spathe length and flower number per male spathe were measured based on three non-dehiscent, mature spathes from each male. Mean ovule number per female spathe was measured based on three non-dehiscent female spathes from each female. Mean fruit length and seed number per fruit were measured based on three mature fruits from each female.

Every plant was partitioned into belowground structures (including roots and stolons), leaves, and sexual reproductive structures (including spathes, peduncles and fruits). The plant parts were dried to constant weight at 80°C and weighed. Considering that some male spathes had withered at the time when the plants were harvested, we randomly sampled 5–10 non-dehiscent, mature male spathes from each male plant within each depth and weighed the mass per spathe. Then we multiplied the mean values of spathe mass obtained at each water depth by the total number of spathes per male to give an estimate of the biomass of sexual reproductive structures of male plants for each water depth. Similarly, we estimated the biomass of mature fruits with intact peduncles of female plants for each water depth. Biomass allocation to reproduction, leaves and belowground structures was determined by dividing the mass of each by the total biomass of the plant. In total, 19 and 20 traits (including photosynthesis) were measured for male and female individuals, respectively (16 was common in both sexes).

### Experiment II: Detection of Sex-Specific Plasticity

Five never-transplanted seedlings were randomly selected from each family (a total of 150 seedlings) to transplant into pots (the same methods as above). The pots were then placed in the greenhouse pool of the Fudan University (the pool water temperature was 20–30°C during the day and 15–25°C at night, with natural light, and a water depth of ∼0.25 m). The growth status of each plant was checked at the beginning of July. From each family, the individual with the most ramets was selected (minimum of nine ramets), and six ramets of similar size were selected to be transplanted into new pots after measuring the length of the longest leaf. Subsequently, three pots of each family were placed at water depths of 0.3 and 1.3 m in the outdoor experimental pool (a total of 90 pots at each water depth). Finally, there were two water depth treatments, two sex treatments, 30 families (17 male and 13 female genotypes) and three replicates per treatment (a total of 102 males and 78 females). The pots at the water depth of 1.3 m were connected by a nylon string to lift them to facilitate the measurement of the plants at any time. A hundred days after transplantation all plants were harvested. Five vegetative traits, six reproductive traits and photosynthetic rate were measured (see Figure 3).

### Determination of Photosynthetic Rate

Photosynthesis was measured both in the first and the second experiment. During the vigorous growth period of *V. natans* (late August to early September), an intact mature leaf was collected from 15 randomly selected males and 15 randomly selected females in each of the water depth treatments (a total of 90 leaves). A segment of each leaf (1/10 of leaf length from the top of each leaf, with a fresh weight of 0.3 g) was cut and incubated in a glass-stoppered tube (3.2-cm diameter, 15-cm length) with pool water to measure light saturated photosynthesis under ambient temperature conditions. Then the tubes were placed under a 3200-lx (∼690 μmol m^–2^ s^–1^) fluorescent tube, with all leaves parallel to the lamp. Another eight glass tubes filled with pool water but without leaf fragment were served as control. The initial dissolved oxygen (DO_0_, mg L^–1^) was measured using JPM-607A electronic dissolved oxygen analyzer (Rex, Shanghai, China), and then all samples, including the controls, were placed under the fluorescent tube (a total of 98 samples). Tubes were moved regularly to make sure that the boundary layer did not cause CO_2_ to become limiting for the plant’s photosynthesis. After 4 h of continuous exposure to irradiation with a constant temperature of 25°C, the leaf fragments were removed. Then the electrode of the dissolved oxygen analyzer was quickly inserted into the glass tube to measure the dissolved oxygen (DO_4h_) of the water sample. The change in dissolved oxygen (ΔDO) of each leaf sample over 4 h was obtained by subtracting the average DO_0_ of the eight control samples. The photosynthetic rate (mg⋅L^–1^ g^–1^ h^–1^) of each leaf sample was obtained by dividing ΔDO by 4 h and 0.3 g (fresh weight of each leaf sample used in the incubation).

### Statistical Analysis

In this study, all of 23 detected phenotypic traits in experiment I were analyzed using one-way ANOVA for sex, water depth, and family. For the effect of depth and family, we analyzed the male and female’s traits separately. For the nine shared traits (percentage of flowering ramets, leaf length, leaf width, spathe number, vegetative biomass, total biomass, reproductive biomass, reproductive effort and photosynthetic rate), the equation that we used for calculating sexual dichotomous index (*SDI*) is *SDI* = Log (*M*: *F*), where *M* and *F* were values of male and female traits, respectively; and the significance of *SDI* was analyzed by χ^2^ tests (to determine whether it is significantly biased toward zero). The differences among water depths and between sexes were compared by a Tukey honestly significant difference (HSD) test procedure (α < 0.05). Two-way ANOVA was performed with depth and sex as independent variables and all the traits in experiment II as dependent variables. If there is an interaction effect, males and females change differently in the given trait with depth. All of the statistical analyses were performed using SPSS 19.0 software (SPSS, Chicago, IL, United States).

## Results

### Sexual Dimorphism in Vegetative and Reproductive Traits

**FIGURE 1:**
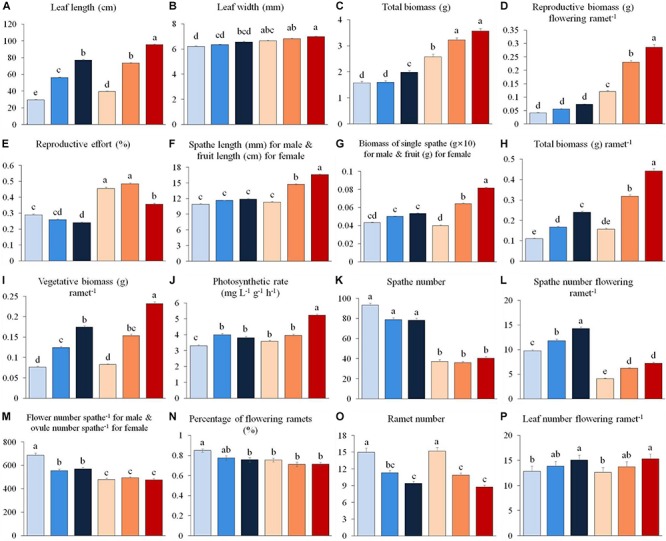
Variation in vegetative and reproductive traits of male (0.3 m: light blue, 0.8 m: blue, 1.3 m: dark blue) and female (0.3 m: pink, 0.8 m: orange, 1.3 m: red) individuals of *Vallisneria natans* at three water depths. **(A)** leaf length; **(B)** leaf width; **(C)** total biomass; **(D)** reproductive biomass per flowering ramet; **(E)** reproductive effort; **(F)** length of male spathe/female fruit; **(G)** biomass of single male spathe/female fruit; **(H)** total biomass; **(I)** vegetative biomass per ramet; **(J)** photosynthetic rate; **(K)** spathe number; **(L)** spathe number per flowering ramet; **(M)** flower number per male spathe and ovule number per female spathe; **(N)** percentage of flowering ramets; **(O)** ramet number; **(P)** leaf number per flowering ramet. Letter superscripts denote significant differences (*P* < 0.05) based on Tukey HSD comparisons. *N* = 90 male and 90 female plants.

We found that 13 out of 16 shared traits differed between the sexes (Table 1 and Figure 1). At all water depths, females displayed higher investment in leaf length, leaf width, total biomass, reproductive biomass per flowering ramet and reproductive allocation compared to males (Figures 1A–E). The vegetative and total biomass per ramet, length of the spathe/fruit (spathe in males and fruit in females) and biomass of the spathe/fruit tended to be greater in females than males at the two higher water depths (Figures 1F–I). Photosynthetic rate was greater in females than in males at both low (0.3 m) and high (1.3 m) water depths (Figure 1J). By contrast, males had a larger amount of spathes, spathes per flowering ramet and gametes per spathe than females across all depths (Figures 1K–M), and exhibited a higher percentage of flowering ramets than females, but only at the low water depth (Figure 1N). There was no difference in ramet number (Figure 1O), leaf number per flowering ramet (Figure 1P) and belowground structure biomass (Table 1) between the sexes irrespective of change in water depth.

**TABLE 1:** One-way ANOVAs for 10 vegetative and 13 reproductive traits of *Vallisneria natans* between sexes, among water depths within sex, and among families within sex.

		**Depth (*df* = 2)**		**Family (*df* = 29)**	
**Trait**	**Sex (*df* = 1)**	**Males**	**Females**	**Males**	**Females**
Leaf length	48.61^∗∗∗^	428.54^∗∗∗^	547.68^∗∗∗^	0.53	0.49
Leaf width	29.35^∗∗∗^	3.23	2.35	1.55^*^	1.87^∗∗^
Total biomass	121.39^∗∗∗^	9.90^*^	16.20^∗∗∗^	2.41^∗∗∗^	2.40^∗∗∗^
Reproductive biomass per flowering ramet	299.42^∗∗∗^	21.47	94.75^∗∗∗^	1.22	0.95
Reproductive effort	292.74^∗∗∗^	6.11^*^	28.39^∗∗∗^	2.00^∗∗^	3.13^∗∗∗^
Male spathe length	–	6.20	–	1.56^*^	–
Fruit length	–	–	105.29^∗∗∗^	–	0.97
Biomass of single male spathe	–	8.49	–	1.05	–
Biomass of single female fruit	–	–	187.27^∗∗∗^	–	0.97
Total biomass per ramet	99.55^∗∗∗^	37.33^∗∗∗^	86.01^∗∗∗^	1.43	0.86
Vegetative biomass per ramet	15.94^∗∗∗^	37.18^∗∗∗^	95.00^∗∗∗^	1.30	1.15
Photosynthetic rate	37.59^∗∗∗^	4.23^*^	79.44^∗∗∗^	4.37^∗∗^	0.27
Spathe number	207.43^∗∗∗^	2.86	1.22	3.11^∗∗∗^	3.44^∗∗∗^
Spathe number per flowering ramet	230.12^∗∗∗^	13.62^∗∗∗^	48.61^∗∗∗^	1.45	1.40
Flower number per male spathe	–	50.47^∗∗∗^	–	2.10^∗∗∗^	–
Ovule number per female spathe	–	–	28.52	–	8.62^∗∗∗^
Percentage of flowering ramets	15.23^∗∗∗^	5.92^∗∗^	1.24	1.36	1.20
Ramet number	0.13	7.77^∗∗^	13.97^∗∗∗^	2.74^∗∗∗^	2.03^∗∗^
Leaf number per flowering ramet	3.17	2.21^*^	0.33^*^	7.18^∗∗∗^	6.64^∗∗^
Fruit number per flowering ramet	–	–	30.54^∗∗∗^	–	1.11
Biomass of belowground structures	0.48	0.14	0.18^∗∗∗^	1.42	1.61^*^
Biomass of leaves	22.77^∗∗∗^	14.12^∗∗∗^	12.85^∗∗∗^	2.24^∗∗^	3.07^∗∗∗^
Biomass of reproductive structures	299.01^∗∗∗^	2.37	17.56^∗∗∗^	2.24^∗∗^	2.06^∗∗^

*F*-values are presented. ^*^P < 0.05; ^∗∗^*P* < 0.01; ^∗∗∗^*P* < 0.001. *df* = degree of freedom. The 16 common traits between males and females are underlined.

### Effects of Water Depth on Male and Female Traits

The leaf length, vegetative and total biomass (per flowering ramet), photosynthetic rate, spathe number per flowering ramet, as well as leaf number per flowering ramet for both sexes increased significantly with increasing water depth (Figures 1A,C,H–J,L,P and Table 1). In females, fruit length increased with increasing water depth (Figure 1F), whereas the number of ovules per spathe did not change with water depth (Figure 1M). By contrast, in males, length and biomass of the spathe remained unchanged across water depths (Figures 1F,G), while the number of flowers per spathe was greater in shallower water (Figure 1M). In both males and females, the reproductive effort was significantly reduced at the high water depth (Figure 1E and Table 1). Family had a significant effect on 11 and 10 traits for males and females, respectively (Table 1).

### Effects of Water Depth on Sexual Dimorphism

**FIGURE 2:**
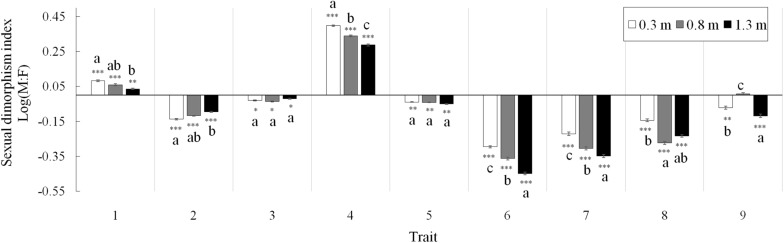
Sexual dimorphism indices of nine shared vegetative and reproductive traits between males and females of *Vallisneria natans* at three water depths. Trait (1) percentage of flowering ramets; (2) leaf length; (3) leaf width; (4) spathe number; (5) biomass of vegetative structures; (6) biomass of reproductive structures; (7) total biomass; (8) reproduction effort; and (9) photosynthetic rate. Asterisks indicate the significance of deviation from 0 (χ^2^ test, ^*^P < 0.05; ^∗∗^*P* < 0.01; and ^∗∗∗^*P* < 0.001). Letter superscripts denote significant differences (*P* < 0.05) among water depths based on Tukey HSD comparisons. *N* = 90 male and 90 female plants.

Both the percentage of flowering ramets and spathe number were male-biased, and sexual differences in both traits decreased with depth (Figure 2; traits 1, 4). The degree of sexual dimorphism in leaf length (female-biased) decreased at higher water depths (Figure 2; trait 2), whereas that in leaf width maintained constant among depths (trait 3). At all water depths, vegetative, reproductive and total biomass and reproductive effort were consistently female-biased (traits 5–8). The degree of sexual dimorphism in biomass of reproductive structures, total biomass and reproduction effort were greater at higher water depths (Figure 2; traits 6–8), whereas sexual difference in vegetative biomass did not change with water depth (trait 5). The degree of sexual dimorphism in photosynthetic rate (female-biased) increased at the water depth of 1.3 m compared to 0.3 m (Figure 2; trait 9).

### Sex-Specific Plastic Responses to Water Depth

**FIGURE 3:**
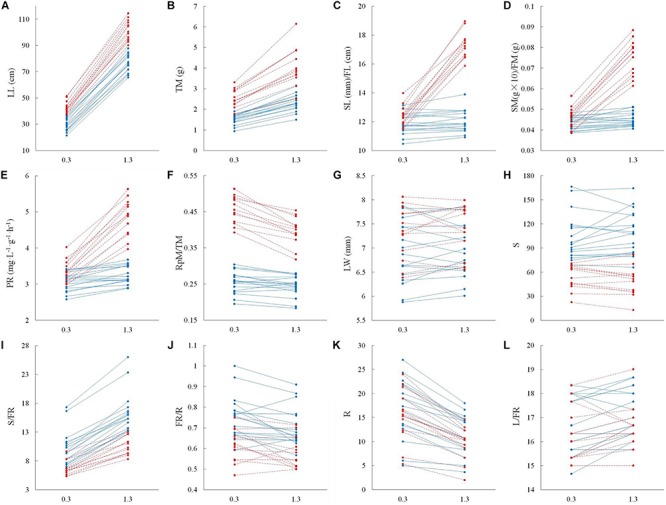
Adaptive strategy in vegetative and reproductive traits in male vs. female individuals of *Vallisneria natans* at 0.3 and 1.3 m water depths. Solid blue lines indicate the 17 male genotypes, and dashed red lines indicate the 13 female genotypes. **(A)** leaf length; **(B)** total biomass; **(C)** length of male spathe/female fruit; **(D)** biomass of single male spathe/female fruit; **(E)** photosynthetic rate; **(F)** reproductive effort; **(G)** leaf width; **(H)** spathe number; **(I)** spathe number per flowering ramet; **(J)** percentage of flowering ramets; **(K)** ramet number; and **(L)** leaf number per flowering ramet.

The significant interactive effect of sex and water depth for 6 out of 12 life history traits that we tested for 17 male and 13 female genotypes indicated that there was substantial intersexual difference in the responses of adaptive traits to water depth change (Table 2). Females generally displayed greater response to variation of water depth than males (Figure 3). Leaf length, total biomass, length of the spathe/fruit, biomass of the spathe/fruit and photosynthetic rate increased more in females than in males at the higher water depth (Figures 3A–E and Table 2). An increase in water depth caused females to reduce their relative allocation to reproduction more than males did (Figure 3F). Leaf width, spathe number, spathe number per flowering ramet, percentage of flowering ramets, ramet number and leaf number per flowering ramet showed no difference in the plastic responses of the two sexes to water depth change (Figures 3G–L and Table 2).

**TABLE 2:** Results of two-way ANOVAs of 12 phenotypic traits for *Vallisneria natans* males (17 genotypes) and females (13 genotypes) grown at 0.3 m and 1.3 m water depths.

		**Source of variation**			
**Dependent variable**	**Results**	**Depth**	**Sex**	**Depth × Sex**	**Error**
		**(*df* = 1)**	**(*df* = 1)**	**(*df* = 1)**	**(*df* = 176)**
Leaf length	MS	40339.91	5493.79	542.75	37.68
	*F*	1070.54	145.79	14.40	
	*P*	**<0.001**	**<0.001**	**<0.001**	
Total biomass	MS	20.22	29.69	2.07	0.29
	*F*	69.88	102.58	7.16	
	*P*	**<0.001**	**<0.001**	**0.010**	
Length of male spathe/female fruit	MS	93.37	124.20	80.26	0.60
	*F*	156.82	208.60	134.79	
	*P*	**<0.001**	**<0.001**	**<0.001**	
Biomass of single male spathe/female fruit	MS	0.003	0.004	0.003	2.68 × 10^–5^
	*F*	129.39	148.29	100.54	
	*P*	**<0.001**	**<0.001**	**<0.001**	
Photosynthetic rate	MS	10.03	13.38	6.10	0.11
	*F*	88.41	118.00	53.81	
	*P*	**<0.001**	**<0.001**	**<0.001**	
Reproductive effort	MS	0.02	0.46	0.009	0.001
	*F*	15.50	403.13	7.75	
	*P*	**<0.001**	**<0.001**	**0.007**	
Leaf width	MS	0.04	2.53	0.001	0.34
	*F*	0.11	7.51	0.004	
	*P*	0.739	**0.008**	0.951	
Spathe number	MS	3.09	45867.76	358.62	573.11
	*F*	0.005	80.03	0.63	
	*P*	0.942	**<0.001**	0.432	
Spathe number per flowering ramet	MS	377.27	294.46	21.72	7.24
	*F*	52.08	40.65	3.00	
	*P*	**<0.001**	**<0.001**	0.089	
Percentage of flowering ramets	MS	0.03	0.27	0.001	0.008
	*F*	3.40	32.84	0.06	
	*P*	0.071	**<0.001**	0.803	
Ramet number	MS	457.98	42.38	2.35	24.90
	*F*	18.39	1.70	0.09	
	*P*	**<0.001**	0.197	0.760	
Leaf number per flowering ramet	MS	2.24	3.58	0.37	1.12
	*F*	2.01	3.20	0.33	
	*P*	0.162	0.079	0.566	

**df* = degree of freedom. Significant results are in bold. Traits with significant depth × sex interaction were underlined.*

## Discussion

Plants of *V. natans* displayed considerable plasticity in growth and reproductive traits in response to water depth change. Although phenotypic plasticity has been the focus of many previous studies on submerged macrophytes (Strand and Weisner, 2001; Riis et al., 2012; Li et al., 2017, 2018), plasticity in sexual dimorphism in the sexes of dimorphic species still remains to be investigated. In our experiments, leaf length, total biomass and leaf photosynthetic rate of males and females increased with increasing water depths (and decreasing light levels), indicating no evidence of light limitation of photosynthesis, which suggested that plants acclimated to the low light conditions (1.3 m deep). While light was reduced with increasing water depth, photosynthetically active radiation determined at the water depth of 0.3 m was very high. Therefore, shallow water was likely to expose the plants to excess light energy at high light intensity (Masini and Manning, 1997; Ruiz and Romero, 2003), potentially causing high light inhibition.

For submerged macrophytes, the efficiency of carbon assimilation is strongly affected by reduced or variable light availability in the water column (Bornette and Puijalon, 2011). It is, therefore, critical for submerged macrophytes to evolve adaptive strategies (e.g., phenotypic plasticity) to reduced light availability (Strand and Weisner, 2001; Fu et al., 2012; Chen et al., 2016). In the present study, sexual morphs of *V. natans* had striking differences in their life history traits, and reproductive males and females differed in how they adjusted their expression of important traits in response to altered water depths with distinct light availability. The patterns of sexual dimorphism in response to different environmental conditions observed in *V. natans* may contribute to maximize each sex’s growth and reproduction in habitats having heterogeneous environments (Sánchez-Vilas et al., 2012).

### Sexual Dimorphism in Growth and Reproduction

Our results show intersexual differences in a wide range of life history traits. Males displayed greater percentage of flowering ramets, and produced more spathes and flowers than females, whereas females displayed a greater resource allocation to reproduction (as a percentage of total biomass in reproductive structures). Many studies have found that females expended proportionally more of their resources on reproduction per reproductive bout than their male counterparts (Delph, 1999; Obeso, 2002; Sakai et al., 2006), including those on the clonal macrophytes *V. americana* (Lovett Doust and Laporte, 1991) and *V. spinulosa* (Li et al., 2019a,b). In comparison to males, females produce fruits and seeds in addition to flowers. Since the energetic requirements for producing fruits are generally greater than for flower production, and the maturation of fruits usually extends for a considerable period after flowering, females must continue to expend resources longer than do males (Sánchez-Vilas et al., 2012). Another possible explanation for the higher reproductive investment in females is that gains in male fitness might be expected to level off with increasing reproductive investment in dioecious hydrophilous species, since the surface of static water can saturate with pollen and the related pollen gains end up competing with one another.

We found that vegetative traits reflecting plant size (leaf length, vegetative biomass, total biomass) and light reception (leaf width, leaf biomass, photosynthetic rate) in females were greater than in males, in spite of the fact that overall fraction of biomass devoted to reproductive organs was much greater in females than males. This suggests that females have a mechanism to compensate by producing more biomass for the extra investment in reproduction. In dioecious *V. natans*, the timing of investment in growth vs. reproduction within a growing season varied between sexes (Zhou et al., 2016). Male growth may be compromised early in the growing season when plants flower and need to produce nitrogen-rich pollen (Harris and Pannell, 2008; Teitel et al., 2016), and this is likely to impact on plant size later in life. Earlier reproductive maturity in males than females has been documented in several dioecious species (Harris and Pannell, 2008; Teitel et al., 2016). In contrast, females are expected to allocate more toward leaf tissue early in the growth season, thus assuring the acquisition of more photosynthates for later allocation to reproduction (higher fecundity) (Delph et al., 1993).

The fact that *V. natans* plants photosynthesized more at higher depths suggests that plants adjust their photosynthetic physiological responses to lower light levels (deep water) to enhance light capturing ability, and this combined with leaf elongation toward the surface of water as a way to alleviate low light stress. Submerged macrophytes growing under low light stress usually increase the light capturing ability of plant leaves (Masini and Manning, 1997; Ruiz and Romero, 2003). It has been reported that *V. natans* has a decreased photosynthetic light compensation point and an increased carbon reservoir under conditions of low light (Yuan et al., 2016). Photosynthesis is increasingly enhanced in females, but not so in males with increasing water depth, suggesting that while light was reduced with increasing water depth, females had higher plasticity in photosynthetic physiological responses compared with males. A possible explanation for this response is that increasing depth reduced photoinhibition (more in females) which resulted in higher net photosynthesis. Sexual dimorphism in growth, resource allocation and photosynthesis across water depths may impact the capacity of males and females for carbon acquisition and thus compete with individuals of different sex.

### Differential Plastic Responses in Males vs. Females

Our previous investigation on the effects of water depth change on reproductive allocation in perennial *V. spinulosa* which produces abundant tubers reported sex-specific plasticity in allocation patterns across a water depth gradient (from 50 to 150 cm) (Li et al., 2019a). Also in the present experiment, males and females of annual *V. natans* differed in adjustments of their morphology, reproductive traits and photosynthesis with increasing water depth. This species relies on the seeds rather than tubers as the main reproductive strategy (Zhang et al., 2016). Thus, our study is useful to determine the extent to which sex-specific plasticity in sex dimorphism is widespread. This finding is consistent with the prediction that adaptive strategies to reduced light availability vary between sexes and provides a possible explanation for observed plasticity in sexual dimorphism across water depths.

In the present experiments, the majority of reproductive traits of observed *V. natans* males and females were responsive to the increase of water depth. Both the number of seeds (0.3 m: 2881 ± 6, 0.8 m: 2600 ± 5, and 1.3 m: 1560 ± 3) and male flowers (0.3 m: 30110 ± 17, 0.8 m: 25741 ± 15, and 1.3 m: 15144 ± 9) that produced per unit of total plant biomass decreased drastically with increasing water depth (*P* < 0.001). This implies a reduction in resources consumed to produce a male flower or a seed at shallower depths. Consistent with our predictions, females had a greater decrease in reproductive allocation than males in response to low light conditions in deep water. First, although light capturing ability (photosynthetic rate) of leaves in females increased more than that in males in the low light, deep water environment as a result of reduced photoinhibition, female reproduction incurs a higher cost than males. This is because flowering in females requires substantial additional investment in flowering bearing structures (very long peduncles) to support flowers to the water surface in deep water (Li et al., 2017). Alternatively, when light became limiting during growth, females may decrease their reproductive allocation more than males, possibly as a way to maintain the future capacity of carbon acquisition structures for future fruit and seed production (Tonnabel et al., 2017). It is therefore, not surprising that increasing water depth and decreasing light levels has greater impacts on the allocation of resources to female reproductive organs.

Males and females differed in their plastic responses in length of the spathe/fruit, biomass of the spathe/fruit, and the number of gametes per spathe/fruit in response to the variation of water depth. In females, fruit length increased with increasing water depth, whereas the number of gametes (ovules) per spathe (or seed number per fruit) did not change with water depth. This response may be due to the need for females to accumulate more pectin and chlorophyll to facilitate fruit photosynthesis and seed maturation in low light, deep water environment, thereby reducing the vegetative burden borne by leaves. There are numerous studies reporting the contribution of fruits to the maintenance their own carbon (Reekie and Bazzaz, 1987; Galen et al., 1993; Imai and Ogawa, 2009). By contrast, in males, length and biomass of the spathe remained unchanged across water depths, while the number of flowers per spathe was greater in shallower water. With average plant height of 34.6 cm at a water depth of 30 cm, it was very likely that leaves on plants in the shallow water treatment clustered on the water surface. Consequently, low water depth is likely to result in spatial clustering of male gametes, which potentially limits pollen dispersal (Lovett Doust and Laporte, 1991) and causes pollen discounting (the reduction in fitness through male function via lost outcross siring opportunities). Selection in male plants will, therefore, optimize the quantity of mating opportunities and improve outcross siring success by enhancing the production of flowers and pollen (Wolf and Takebayashi, 2004; Charlesworth, 2006).

Contrary to our predictions that the sexual dimorphism in vegetative investment increases in more stressful environments, size differences in *V. natans*, in terms of vegetative biomass, were not greatly affected by water depth. However, reduced light availability resulted in a greater increase in leaf length, total biomass and photosynthetic rate in females than in males.

Females should maximize allocation to aboveground growth under conditions of light attenuation more than should males, in combination with a stronger reduction of allocation to reproductive tissues, because carbon acquisition is more critical for female reproduction (higher fecundity) (Tonnabel et al., 2017). The effect of sex-specific reaction to underwater light availability is that females conserve relatively more capacity for future carbon acquisition than males, thus ensuring their future carbon-rich reproduction. At the same time, males would benefit of smaller size due to the advantage of earlier maturation with related demographic advantages of precocious reproduction (Andersson, 1994). Indeed, environmental heterogeneity (e.g., floods, eutrophication, resuspension of soft surface sediment and increases in periphyton) can strongly affect access to light for submerged macrophytes in natural waterbodies. If light limitation elicits sex-specific plasticity, then spatial variation in light availability will bring about spatial variation in the degree of sexual dimorphism or sex ratios among populations of a species. The sex-specific plastic response in *V. natans* to water depth change implies that growth and reproduction of male and female individuals can be affected differently by flooding and water level fluctuations in freshwater habitats, producing variations in the degree of sexual dimorphism. Consequently, long-distance dispersal and colonization of submerged dioecious macrophytes may be impacted by environmental variations in aquatic ecosystems. More studies across aquatic dioecious species are required to make general conclusions on the effects of altered environmental conditions on growth and reproduction of aquatic vegetation via adaptive strategies, e.g., plasticity in sexual dimorphism.

## Author Contributions

YZ and ZS designed the research. YZ conducted the experiments. LL and YZ conducted the statistical analysis. LL wrote the manuscript. All authors commented on and approved the final draft of the manuscript.

## Conflict of Interest Statement

The authors declare that the research was conducted in the absence of any commercial or financial relationships that could be construed as a potential conflict of interest.

## References

[B1] Andersson M. (1994). *Sexual Selection.* Princeton, NJ: Princeton University Press.

[B2] Barrett S. C. H. Hough J. (2013). Sexual dimorphism in flowering plants. *J. Exp. Bot.* 64 67–82. 10.1093/jxb/ers308 23183260

[B3] Bornette G. Puijalon S. (2011). Response of aquatic plants to abiotic factors: a review. *Aquat. Sci.* 73 1–14. 10.1007/s00027-010-0162-7 15356219

[B4] Chambers P. A. Kaiff J. (1985). Depth distribution and biomass of submersed aquatic macrophyte communities in relation to Secchi depth. *Can. J. Fish. Aquat. Sci.* 42 701–709. 10.1139/f85-090

[B5] Charlesworth D. (2006). Evolution of plant breeding systems. *Curr. Biol.* 16 R726–R735. 10.1016/j.cub.2006.07.068 16950099

[B6] Chen J. F. Cao T. Zhang X. L. Xi Y. L. Ni L. Y. Jeppesen E. (2016). Differential photosynthetic and morphological adaptations to low light affect depth distribution of two submersed macrophytes in lakes. *Sci. Rep.* 6:34028. 10.1038/srep34028 27694880 PMC5046178

[B7] Cornelissen T. Stiling P. (2005). Sex-biased herbivory: a meta-analysis of the effects of gender on plant-herbivore interactions. *Oikos* 111 488–500. 10.1111/j.1600-0706.2005.14075.x

[B8] Dawson T. E. Ehleringer J. R. (1993). Gender-specific physiology, carbon isotope discrimination, and habitat distribution in box elder *Acer negundo*. *Ecology* 74 798–815. 10.2307/1940807

[B9] Dawson T. E. Geber M. A. (1999). “Sexual dimorphism in physiology and morphology,” in *Gender and Sexual Dimorphism in Flowering Plants*, eds Geber M. A. Dawson T. E. Delph L. F. (Berlin: Springer-Verlag), 175–215. 10.1007/978-3-662-03908-3_7

[B10] Delph L. E. Lu Y. Jayne L. D. (1993). Patterns of resource allocation in a dioecious *Carex* (Cyperaceae). *Am. J. Bot.* 80 607–615. 10.2307/2445429

[B11] Delph L. F. (1999). “Sexual dimorphism in life history,” in *Gender and Sexual Dimorphism in Flowering Plants*, eds Geber M. A. Dawson T. E. Delph L. F. (Berlin: Springer-Verlag), 149–174.

[B12] Delph L. F. Bell D. L. (2008). A test of the differential-plasticity hypothesis for variation in the degree of sexual dimorphism in *Silene latifolia*. *Evol. Ecol. Res.* 10 61–75. 10.1159/000084019 15722629

[B13] Dorken M. E. Barrett S. C. (2004). Phenotypic plasticity of vegetative and reproductive traits in monoecious and dioecious populations of *Sagittaria latifolia* (Alismataceae): a clonal aquatic plant. *J. Ecol.* 92 32–44. 10.1111/j.1365-2745.2004.00857.x

[B14] Eckert C. G. Dorken M. E. Barrett S. C. H. (2016). Ecological and evolutionary consequences of sexual and clonal reproduction in aquatic plants. *Aquat. Bot.* 135 46–61. 10.1016/j.aquabot.2016.03.006 26195747

[B15] Eckhart V. M. Chapin F. S. (1997). Nutrient sensitivity of the cost of male function in gynodioecious *Phacelia linearis* (Hydrophyllaceae). *Am. J. Bot.* 84 1092–1098. 10.2307/2446152 21708664

[B16] Fu H. Yuan G. X. Cao T. Ni L. Y. Zhang M. Wang S. R. (2012). An alternative mechanism for shade adaptation: implication of allometric responses of three submersed macrophytes to water depth. *Ecol. Res.* 27 1087–1094. 10.1007/s11284-012-0991-z

[B17] Fu H. Zhong J. Y. Yuan G. X. Xie P. Guo L. G. Zhang X. L. (2014). Trait-based community assembly of aquatic macrophytes along a water depth gradient in a freshwater lake. *Freshwater Biol.* 59 2462–2471. 10.1111/fwb.12443

[B18] Galen C. Dawson T. E. Stanton M. L. (1993). Carpels as leaves: meeting the carbon cost of reproduction in an alpine buttercup. *Oecologia* 95 187–193. 10.1007/BF00323489 28312941

[B19] Geber M. A. Dawson T. E. Delph L. F. (1999). *Gender and Sexual Dimorphism in Flowering Plants*, 1st Edn Heidelberg: Springer.

[B20] Harris M. S. Pannell J. R. (2008). Roots, shoots and reproduction: sexual dimorphism in size and costs of reproductive allocation in an annual herb. *P. Roy. Soc. B Biol. Sci.* 275 2595–2602. 10.1098/rspb.2008.0585 18682371 PMC2605799

[B21] Hesse E. Pannell J. R. (2011). Sexual dimorphism in a dioecious population of the wind-pollinated herb *Mercurialis annua*: the interactive effects of resource availability and competition. *Ann. Bot. London* 107 1039–1045. 10.1093/aob/mcr046 21385775 PMC3080628

[B22] Hultine K. R. Grady K. C. Shuster S. M. Stella J. C. Whitham T. G. (2016). Climate change perils for dioecious plant species. *Nat. Plants* 2:16109. 10.1038/nplants.2016.109 28221374

[B23] Imai S. Ogawa K. (2009). Quantitative analysis of carbon balance in the reproductive organs and leaves of *Cinnamomum camphora* (L.) Presl. *J. Plant Res.* 122 429–437. 10.1007/s10265-009-0233-9 19396511

[B24] Les D. H. (1988). Breeding systems, population structure, and evolution in hydrophilous angiosperms. *Ann. Miss. Bot. Gard.* 75 819–835.

[B25] Li L. Barrett S. C. H. Song Z. P. Chen J. K. (2019a). Sex-specific plasticity of reproductive allocation in response to water depth in a clonal, dioecious macrophyte. *Am. J. Bot.* 106 42–50. 10.1002/ajb2.1218 30629301

[B26] Li L. Ding M. M. Lan Z. C. Zhao Y. Chen J. K. (2019b). Light Availability and patterns of allocation to reproductive and vegetative biomass in the sexes of the dioecious macrophyte *Vallisneria spinulosa*. *Front. Plant Sci.* 10:572. 10.3389/fpls.2019.00572 31130977 PMC6510307

[B27] Li L. Bonser S. P. Lan Z. C. Xu L. G. Chen J. K. Song Z. P. (2017). Water depth affects reproductive allocation and reproductive allometry in the submerged macrophyte *Vallisneria natans*. *Sci. Rep.* 7:16842. 10.1038/s41598-017-16719-1 29203795 PMC5715065

[B28] Li L. Lan Z. C. Chen J. K. Song Z. P. (2018). Allocation to clonal and sexual reproduction and its plasticity in *Vallisneria spinulosa* along a water-depth gradient. *Ecosphere* 9:e02070 10.1002/ecs2.2070

[B29] Lovett Doust J. Laporte G. (1991). Population sex ratios, population mixtures and fecundity in a clonal dioecious macrophyte. *Vallisneria americana*. *J. Ecol.* 79 477–489. 10.2307/2260727

[B30] Maberly S. C. (1993). Morphological and photosynthetic characteristics of *Potamogeton obtusifolius* from different depths. *J. Aquat. Plant Manage.* 31 34–39.

[B31] Masini R. J. Manning C. R. (1997). The photosynthetic responses to irradiance and temperature of four meadow-forming seagrasses. *Aquat. Bot.* 58 21–36. 10.1016/S0304-3770(97)00008-9

[B32] Nicotra A. B. (1999). Reproductive allocation and the long-term costs of reproduction in *Siparuna grandiflora*, a dioecious neotropical shrub. *J. Ecol.* 87 138–149.

[B33] Obeso J. R. (2002). The costs of reproduction in plants. *New Phytol.* 155 321–348. 10.1046/j.1469-8137.2002.00477.x 33873312

[B34] Pickering C. M. Hill W. (2002). Reproductive ecology and the effect of altitude on sex ratios in the dioecious herb *Aciphylla simplicifolia* (Apiaceae). *Aust. J. Bot.* 50 289–300. 10.1071/BT01043

[B35] Quinn J. A. (1991). Evolution of dioecy in *Buchloe dactyloides* (Gramineae): test for sex-specific vegetative characters, ecological differences, and sexual niche-partitioning. *Am. J. Bot.* 78 481–488. 10.1002/j.1537-2197.1991.tb15214.x

[B36] Reekie E. G. Bazzaz F. A. (1987). Reproductive effort in plants. I. Carbon allocation to reproduction. *Am. Nat.* 129 876–896. 10.1086/284681

[B37] Riis T. Olesen B. Clayton J. S. Lambertini C. Brix H. Sorrell B. K. (2012). Growth and morphology in relation to temperature and light availability during the establishment of three invasive aquatic plant species. *Aquat. Bot.* 102 56–64. 10.1016/j.aquabot.2012.05.002

[B38] Ruiz J. M. Romero J. (2003). Effects of disturbances caused by coastal constructions on spatial structure, growth dynamics and photosynthesis of the seagrass *Posidonia oceanica*. *Mar. Pollut. Bull.* 46 1523–1533. 10.1016/j.marpolbul.2003.08.021 14643778

[B39] Sakai A. Sasa A. Sakai S. (2006). Do sexual dimorphisms in reproductive allocation and new shoot biomass increase with an increase of altitude? A case of the shrub willow *Salix reinii* (Salicaceae). *Am. J. Bot.* 93 988–992. 10.3732/ajb.93.7.988 21642163

[B40] Sánchez-Vilas J. Bermúdez R. Retuerto R. (2012). Soil water content and patterns of allocation to below- and above-ground biomass in the sexes of the subdioecious plant Honckenya peploides. *Ann. Bot.* 110 839–848. 10.1093/aob/mcs157 22782243 PMC3423814

[B41] Strand J. A. Weisner S. E. (2001). Morphological plastic responses to water depth and wave exposure in an aquatic plant (*Myriophyllum spicatum*). *J. Ecol.* 89 166–175. 10.1046/j.1365-2745.2001.00530.x

[B42] Teitel Z. Pickup M. Field D. L. Barrett S. C. H. (2016). The dynamics of resource allocation and costs of reproduction in a sexually dimorphic, wind-pollinated dioecious plant. *Plant Biol.* 18 98–103. 10.1111/plb.12336 25865555

[B43] Tonnabel J. David P. Pannell J. R. (2017). Sex-specific strategies of resource allocation in response to competition for light in a dioecious plant. *Oecologia* 185 675–686. 10.1007/s00442-017-3966-5 29043498 PMC5681607

[B44] Wolf D. E. Takebayashi N. (2004). Pollen limitation and the evolution of androdioecy from dioecy. *Am. Nat.* 163 122–137. 10.1086/380493 14767842

[B45] Ye B. B. Chu Z. S. Wu A. P. Hou Z. Y. Wang S. R. (2018). Optimum water depth ranges of dominant submersed macrophytes in a natural freshwater lake. *PLoS One* 13:e0193176. 10.1371/journal.pone.0193176 29513707 PMC5841742

[B46] Yuan G. X. Fu H. Zhong J. Y. Luo Q. Ni L. Y. Cao T. (2016). Growth and C/N metabolism of three submersed macrophytes in response to water depths. *Environ. Exp. Bot.* 122 94–99. 10.1016/j.envexpbot.2015.09.009

[B47] Zhang Y. L. Liu X. H. Qin B. Q. Shi K. Deng J. M. Zhou Y. Q. (2016). Aquatic vegetation in response to increased eutrophication and degraded light climate in eastern lake taihu: implications for lake ecological restoration. *Sci. Rep.* 6:23867. 10.1038/srep23867 27041062 PMC4819184

[B48] Zhou N. N. Hu W. P. Deng J. C. Zhu J. Xu W. W. Liu X. (2017). The effects of water depth on the growth and reproduction of *Potamogeton crispus* in an in situ experiment. *J. Plant Ecol.* 10 546–558. 10.1093/jpe/rtw048

[B49] Zhou Y. Li X. J. Zhao Y. Zhou W. Li L. Wang B. (2016). Divergences in reproductive strategy explain the distribution ranges of *Vallisneria* species in China. *Aquat. Bot.* 132 41–48. 10.1016/j.aquabot.2016.04.005

